# The role of Dynamic Energy Budgets in conservation physiology

**DOI:** 10.1093/conphys/coab083

**Published:** 2021-10-25

**Authors:** Romain Lavaud, Ramón Filgueira, Starrlight Augustine

**Affiliations:** 1 School of Renewable Natural Resources, Louisiana State University Agricultural Center, Baton Rouge, LA 70803, USA; 2 Marine Affairs Program, Dalhousie University, Halifax, Nova Scotia B3H 4R2, Canada; 3 Akvaplan-niva, Fram High North Research Centre for Climate and the Environment, Tromsø 9296, Norway

**Keywords:** species, modelling, metabolic organization, DEB theory, bioenergetics

## Abstract

The contribution of knowledge, concepts and perspectives from physiological ecology to conservation decision-making has become critical for understanding and acting upon threats to the persistence of sensitive species. Here we review applications of dynamic energy budget (DEB) theory to conservation issues and discuss how this theory for metabolic organization of all life on earth (from bacteria to whales) is well equipped to support current and future investigations in conservation research. DEB theory was first invented in 1979 in an applied institution for environmental quality assessment and mitigation. The theory has since undergone extensive development and applications. An increasing number of studies using DEB modelling have provided valuable insights and predictions in areas that pertain to conservation such as species distribution, evolutionary biology, toxicological impacts and ecosystem management. We discuss why DEB theory, through its mechanistic nature, its universality and the wide range of outcomes it can provide represents a valuable tool to tackle some of the current and future challenges linked to maintaining biodiversity, ensuring species survival, ecotoxicology, setting water and soil quality standards and restoring ecosystem structure and functioning in a changing environment under the pressure of anthropogenic driven changes.

## Teaser

This paper reviews applications of dynamic energy budget (DEB) theory to conservation issues. We discuss how the DEB framework, through its mechanistic nature, its universality and the wide range of outputs it provides, is well equipped to tackle current and future challenges in the conservation of species.

## Introduction

Species biodiversity is essential for the thriving of human societies and for our planet’s ecosystems. However, human activities themselves are causing rapid and global changes that are driving biodiversity loss, as well as climate change and land degradation. Urgent, integrated action is thus mandated to preserve the functional role of biodiversity in creating sustainable environments and societies (Brondizio *et al.*, 2019; CBD UNEP, 2019). As time is running out maintaining and restoring biodiversity using a case-by-case approach for each species is unlikely to support achieving these goals (Franklin, 1993; Lindenmayer *et al.*, 2007). Additionally, given the variety of stressors and their potential interactions, mechanistic approaches that rely on unified principles, rather than empirical approaches, should facilitate extrapolating knowledge from species to species (Denny and Helmuth, 2009; Boult and Evans, 2021). Research in ecophysiology and metabolic theories provide the fundamental knowledge that explains the functioning of organisms, populations and ecosystems under the full range of environmental conditions (Cooke *et al.*, 2013; Kearney *et al.*, 2021). Such process-based frameworks should, therefore, help predict, plan for and possibly mitigate the effects of climate change and human pressures on the biology of species through insightful cause-and-effect tools to facilitate decision-making process.

The dynamic energy budget (DEB) theory is a formal metabolic theory of life that presents these attributes (Sousa *et al.*, 2008). This theory describes the uptake of energy from the environment by an organism (feeding and digestion) and the use of this energy for maintenance, development, growth and reproduction throughout the life cycle (Kooijman, 2010). It relies on the concepts of stoichiometry, homeostasis, energy dissipation and the similar metabolic organization of cells among living organisms to build energy budgets for virtually all species. The unprecedented level of generalization and formalism of this theory originates from the guiding principle that the mechanisms responsible for the organization of metabolism apply universally to the organisms of all species (Jusup *et al.*, 2017). Through the standard use of energetic units (e.g. Joules, J), combined with standardized metabolic parameters, comparisons across the organizational (Morgan Ernest and Brown, 2001; Murphy *et al.*, 2018), spatial (Monaco and McQuaid, 2018; Thomas and Bacher, 2018) and temporal scales (Martin *et al.*, 2013) become possible. DEB models now exist for about 3000 species and counting (AmP, 2021) and the number of publications including DEB concepts is exponentially rising (>1000 as of 27 August 2021). Other approaches exist to evaluate physiological performances of organisms and models of various aspects of metabolism have been proposed. These include net production frameworks such as the Scope for Growth (Warren and Davies, 1967) or Wisconsin models (Winberg, 1956; Deslauriers *et al.*, 2017), the Gill-Oxygen Limitation Theory (Pauly, 1979), or the Metabolic Theory of Ecology (West *et al.*, 2001). Comparing DEB theory to these alternatives is outside of the scope of this review but critical evaluations have been published (van der Meer, 2006a; Kearney, 2020).

A small set of DEB parameters and equations allows predicting a variety of life-history traits (lifespan, growth, fertility, physiological rates and tolerance to toxicants, among others) and idealized population level traits (Kooijman *et al.*, 2020). A full definition of the principles governing the standard DEB model can be found in the literature (van der Meer, 2006b; Sousa *et al.*, 2008; Kooijman, 2010; Jusup *et al.*, 2017) as well as methods for estimating parameters (Lika *et al.*, 2011; Marques *et al.*, 2019; Filgueira *et al.*, 2021). A succinct description is given hereafter. The life cycle is divided into three stages: embryo, juvenile and adult. Embryos do not assimilate food, nor reproduce, but allocate energy to maturation. Juveniles start to assimilate, continue to mature, but there is still no reproduction. In adults, assimilation continues, and the energy previously allocated to maturation now fuels the reproduction buffer. Four state variables describe the organism: structure, reserve, maturity and reproduction buffer. Energy fluxes between these variables can be described as follows: at birth, juveniles start acquiring food through a feeding flux proportional to the structural area. Ingested food is transformed and stored in the reserve via an assimilation flux (proportional to the feeding flux) and a faeces product is released. Reserve is used to fuel metabolic needs and unlike structure does not require maintenance. Reserve dynamics is governed by the difference between assimilation and mobilization, which can be computed from first principles (see Sousa *et al.*, 2010). Mobilized reserve is split into two fractions: κ for the somatic branch and (1–κ) for the maturity and reproduction branch. In the somatic branch, somatic maintenance for the existing structure is paid first and what is left is used for growth, the increase of structure. The dynamics of structure is given by the growth flux multiplied by the volume-specific costs for structure. In the maturity and reproduction branch priority goes to pay maturity maintenance proportionally to the existing maturity. The remaining fraction is used as a maturation flux in the embryo and the juvenile stages or as a reproduction flux in adults. The dynamics of maturity is given by the accumulation of energy from the maturation flux. At puberty, the organism reaches maximum maturity, and energy is now allocated to the reproduction buffer. Extensions or deviations from the standard DEB model are considered in the theory to account for specific traits of certain taxa (Kooijman, 2010; Lika and Kooijman, 2011).

All applications of model come with knowledge of parameter values, and great attention has been given over time to software and methodologies for extracting DEB parameters from data (Augustine and Kooijman, 2019). The analysis of laboratory and field data using DEB models and the cycle of improvements of both model and data collection (see the empirical cycle, Figure 1 in Kooijman, 2018) creates a synergy that can support conservation research, as it has supported ecotoxicological research since the late 70s. Indeed, the multi-disciplinary nature of conservation research can benefit from this universal biology-inspired mathematical interface between observations and interpretations. The detailed knowledge of an organism’s metabolism provided through DEB modelling may facilitate the creation of indices of physiological stress and condition, allowing for better estimates of species persistence under different scenarios (e.g. land-use change, climate change and food-web disturbance) and, as such, contribute to the development of conservation physiology (Cooke *et al.*, 2013).

**Figure 1:**
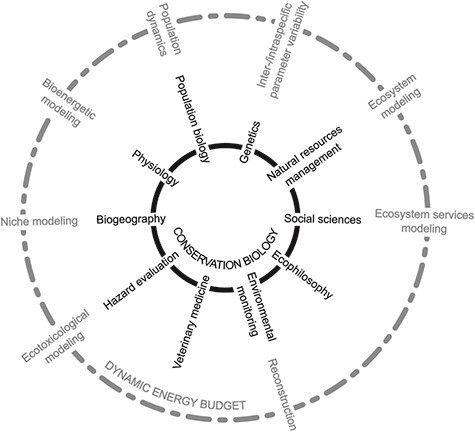
Conceptual diagram aligning DEB modelling frameworks with fields of conservation biology identified by Kareiva and Marvier (2012). Some model applications may be relevant to several DEB modelling frameworks: tipping point studies, for instance, may fall under IBM, niche or ecotoxicological modelling approaches.

As a sign of its universality, the scope of applications of DEB theory has diversified since it was first formalized in 1979, from ecotoxicology, to population dynamics, ecology, diversity and distribution of species, impacts of climate change and many more (Fig. 1). The upcoming special issue in *Conservation Physiology* will seek contributions inspired by the *Seventh International Symposium on DEB theory: Forecasting in a Changing World* (24–28 May 2021), which represents an opportunity for outstanding DEB research to contribute to the growing field of conservation physiology. In this paper, we dive into the DEB literature to survey past and current developments that illustrate the relevance of DEB theory to the field of conservation physiology.

## Diversity and distribution of species

### **Modelling** the diversity of life

**Figure 2:**
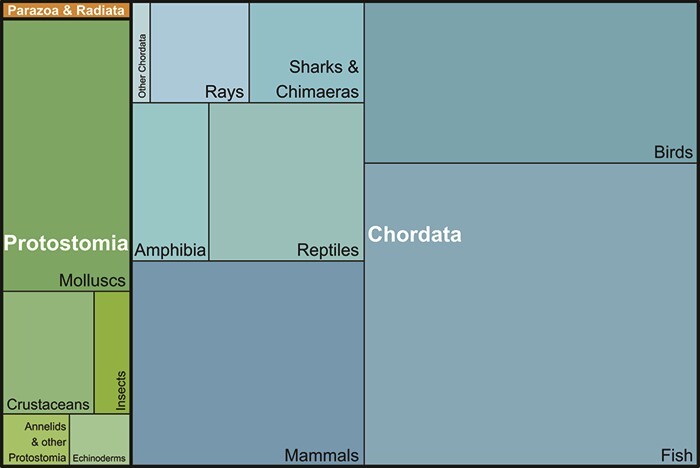
Tree map of the proportion of major taxa in the AmP collection of DEB parameters (as of 27 August 2021).

The conservation of species diversity relies on many factors and in a world that sees increasing pressures from human activities on ecosystems via climate change, pollution, land use, etc. it is paramount to rely on a framework that can integrate these pressures and allows the quantification of these impacts on species through different scales of time and space. Because such an integration requires direct links between the physiological response to these pressures at the cellular or sub-cellular level and higher levels of organization (organism, population, etc.), putting individual metabolic processes at the core of a framework as in DEB theory seems most appropriate to evaluate the fate of different species in different and changing environments. While DEB theory does not provide tools to estimate species richness or diversity indices, the fact that it relies on the empirical evidence of the universality of metabolic processes among organisms makes it applicable to any animal or vegetal species and allows comparison between the traits and model characteristics of these species, which should be useful for conservation efforts. DEB models have been developed for bacteria (Eichinger *et al.*, 2009), micro-algae (Muller and Nisbet, 2014; Livanou *et al.*, 2019) and macro-algae (Lavaud *et al.*, 2020), rotifers (Shertzer and Ellner, 2002), bivalves (Cardoso *et al.*, 2006; Montalto *et al.*, 2015), annelids (De Cubber *et al.*, 2019), insects (Llandres *et al.*, 2015; Maino and Kearney, 2015), sea stars (Monaco *et al.*, 2014; Agüera *et al.*, 2015), sea cucumbers (Ren *et al.*, 2017), fishes (van der Veer *et al.*, 2001; Kooijman and Lika, 2011; Augustine *et al.*, 2017), amphibians (Mueller *et al.*, 2012), lizards (Kearney, 2012; Schwarzkopf *et al.*, 2016), turtles (Marn *et al.*, 2019), birds (Teixeira *et al.*, 2014; Kooijman, 2020a) and mammals (Desforges *et al.*, 2019; Silva *et al.*, 2020). The AmP collection, a database of all DEB parameter sets (as well as underlying data and code) for animals, includes all large phyla (Fig. 2). Chordates are complete at the order level, and primates at the family level (AmP, 2021).

The application of DEB models requires knowledge of parameter values, which determine the processes responsible for the variety of life-history traits observed among species. Estimating DEB parameters for a given species requires data that can be of very different nature, such as the following: length, weight and age at key stages of the life cycle; growth or fertility trajectories through time; and physiological rates (feeding, respiration, excretion, growth, reproduction, etc.) at different temperature or food concentrations. With the development of tools to standardize the procedure for parameter estimation (Lika *et al.*, 2011; Marques *et al.*, 2018), and the increasing number of entries in the AmP database, comparisons between species become more evident. Comparisons over standardized parameters that relate to differences in life-history traits represent a great tool for conservation purposes. Indeed, advocates of a comparative approach to conservation (i.e. phylogenetic comparisons) suggest that it could reveal general mechanisms in conservation, provide shortcuts for prioritizing conservation research and enable us to predict which species will experience (or create) problems in the future (Fisher and Owens, 2004). Ultimately, this approach may help identifying species that are more vulnerable to particular environmental changes based on the dynamics of their energy budget. For instance, Thomas and Bacher (2018) used DEB models to study the changes in distribution and abundance of three species of bivalves (the blue mussel, *Mytilus edulis*; the Pacific oyster, *Crassostrea gigas*; and the flat oyster *Ostrea edulis*) along the Northeast Atlantic coast of Europe under scenarios of climate change. Using individual-based modelling (IBM), the authors identified the reproductive phenology as a core process driving the responses of the populations and emphasized the close relationship between the predicted patterns and the species thermal tolerances. The use of functional traits through metabolic theories may also help initiatives to reference trait data into biodiversity databases, addressing a need to develop a better predictive capacity for how species respond to environmental change (Kearney *et al.*, 2021).

In direct link with conservation considerations, Teixeira (2016) built DEB models for 40 bird species, representing the existing diversity in terms of phylogeny, distribution and life-history traits. By looking into the diversity of DEB parameters, functions and implied properties according to different ecological factors, Teixeira (2016) identified patterns in life-history and bioenergetic traits helping to understand some fundamental energetic trade-offs described in bird species. For instance, this work suggests that for birds breeding in remote places and foraging over large areas, energy reserves partially contribute to reducing the risk from stochasticity in food provisioning during chick development. In a detailed study of the Wandering Albatross, Teixeira *et al.* (2014) further suggested that different behavioural profiles in terms of locomotion effort, before and after fledging, may provide chicks of semi-altricial species with an energy surplus that can be stored in reserve. When colonizing remote breeding sites, these reserves would mitigate the stochasticity related to feeding strategies and increase the chances of survival after fledging, when flying and foraging skills are being learned.

Other examples of inter-specific comparisons of life-history traits through DEB modelling have been published on fish (van der Veer *et al.*, 2001; Kooijman and Lika, 2013), bivalves (Cardoso *et al.*, 2006; van der Veer *et al.*, 2006) and turtles (Marn *et al.*, 2019). The comparison of DEB parameters or variables can shed a new light on shared traits or differences between species and how environmental changes such as an increase in temperatures might impact species distribution (Kearney, 2012), or how changes in food availability might impact interactions between native and invasive species (Thomas *et al.*, 2016).

### Niche modelling

The performance of organisms is tightly linked to the physical environment they live in through their tolerance to the variations in these conditions. This describes the concept of *ecological niche*, which has long been a critical tool in conservation (Cole *et al.*, 1995; Peterson and Robins, 2003). By capturing the metabolic processes of an organism through its entire life cycle, DEB theory provides a unique framework that adds a dynamic dimension to the field of niche modelling, which has usually incorporated phenomenological, static energy budgets and empirical descriptions of physiological processes (snapshot in time; Kearney, 2012). Ectotherms such as reptiles are particularly sensitive to changes in their surrounding environment as they have limited to no control over their internal temperature. A powerful tool recently developed by Kearney and Porter (2020) allows the mechanistic modelling of heat, water, energy and mass exchange between any kind of ectothermic organism and its environment, as well as the inclusion of behavioural options (posture and colour change, shade-seeking, panting, climbing and retreating underground). Despite the level of detail in this application of niche modelling, the applicability of the method is broad and is currently being adapted to endotherms (Kearney *et al.*, 2021).

The study of the distribution of invasive species is another relevant aspect of conservation. Sarà *et al.* (2013) coupled a biophysical ecology model to a DEB model to examine the physiological performance (maximal size and reproductive output) of the invasive mussel, *Brachidontes pharaonic*, in the Mediterranean Sea through the analysis of their fundamental niche. They showed that subtidal sites in the Central Mediterranean are generally suitable for this mussel but that intertidal habitats appear to serve as genetic sinks, which can determine the potential distribution of this invasive species.

### Tipping points

Because DEB theory is based on the conservation of mass and energy, the quantification of metabolic processes allows the identification of tipping points in the ontogeny of a species or the state of system. The dynamic nature of DEB models is also particularly relevant for conservation studies dealing with ever-changing ecosystems and the movement of populations and species. Using an approach based on DEB modelling, Filgueira *et al.* (2016) showed that the proportion of modified land use of the watershed (agricultural and urban land) can reach a tipping point beyond which the functioning of the stream abruptly changes due to changes in stream fish diversity and size-at-age of a generalist fish species. In another example, Marn *et al.* (2020) studied the impacts of plastic debris on the energy budget of marine wildlife and showed that the estimated plastics ingestion was insufficient to impede sexual maturation but may still be responsible for population declines.

Carrying capacity studies can also help identify potential tipping points in the functioning of ecosystems. These ecosystem-level models require coupling the most relevant physical, chemical and biological components and processes of a system (e.g. primary production, food availability, toxicant concentration). Carrying capacity analyses may then estimate the impact of deforestation, fishing activities, farming practices (both inland and in coastal waters) or urban development on an ecosystem. In the context of DEB modelling, most carrying capacity studies were conducted in relation to aquaculture development. Filgueira *et al.* (2014) and later Lavaud *et al.* (2020) designed and coupled a series of DEB models to estimate the impact of current and projected mussel aquaculture on the carrying capacity of coastal bays in Eastern Canada. These studies determined that adding mussel farms would be sustainable when considering primary production, the nutrient cycling equilibrium, the fitness of native bivalve and the risk of eutrophication. Pete *et al.* (2020) determined that the efficiency of wastewater treatment plants (WWTP) in the Thau lagoon (France) affected oyster production without significantly improving the ecological status of the lagoon. Furthermore, oyster production might be threatened by drastic changes to WWTP in the area, specifically through impacts on phosphorus cycling, which seems to control primary production in the Thau ecosystem. These approaches are important for the conservation, planning and management of both resources. The mechanistic description via DEB models of ecosystem-level processes that determine the interactions between the different components of a system constitutes a reliable approach in understanding and quantifying the carrying capacity and potential tipping points of such system.

### Reconstruction of food and physiological histories

Some species living in remote environments or spending periods of their life cycle in areas that are not easily accessible for research data collection are evidently more complex to monitor and protect. The lack of data often represents a major limiting factor to conservation efforts as managing a resource, a population or an ecosystem implies a quantification of its status through time. A particular application of DEB modelling offers an interesting way out of this problem: model inversion. Because DEB models are budgets and are based on set processes that determine energy allocation, it is possible to use what is usually considered an output of the model, such as growth trajectories, to back-track the energy allocation and in some cases infer environmental conditions that usually force the model. A first example of such application is the use of anchovy otoliths and their optical properties to reconstruct food histories (Pecquerie *et al.*, 2012). Reconstructing food conditions of past and present aquatic species in their natural environment provides key ecological information that can be used to better understand environmental impacts on population dynamics and the fate of species. Another example was provided by Lavaud *et al.* (2019a,b) to reconstruct the food history and physiological status (reserve dynamics, maintenance costs, etc.) of scallops along a latitudinal and bathymetric gradient using shell growth increments. From a conservation perspective, understanding what metabolic process (e.g. reserve status, reproductive capacity, maintenance costs) may explain the success or failure of a conservation program without requiring a large amount of data often difficult to obtain. These techniques make use of the internal archive that carbonate structure represent and do not require live animals. Overall, studies on the energetics of species may thus contribute to fill in the gaps of knowledge regarding a large number of poorly documented species.

Contaminants can be present in very low levels in the environment, and sentinel species like the blue mussel can be monitored in order to measure the amount of xenobiotic in their body tissue, indicating a pollution problem. The DEB model has been applied to reconstruct environmental concentrations of pollutants that the mussels would have experienced (van Haren *et al.*, 1994). In another study, Sadoul *et al.* (2019) looked at how exposure to Bisphenol A during the egg phase of rainbow trout impairs the growth of adult fish. The authors reconstructed the stress upon metabolism based on observed changes in growth. These studies represent a promising venue of applying DEB theory to biological time series (condition, spawning, fat content, body residues) in order to reconstruct exposure to stressors, which leads us to our next section.

## Stress ecology and ecotoxicology

### DEBtox models

One of the impacts of humans on natural systems is through changes in the chemistry of the environment, either through the excessive release of chemicals that pollute the water, soil and air, or the perturbation of nutrient cycles causing complex issues such as eutrophication. With a growing human population, stress ecology and ecotoxicology are tightly linked to conservation issues, as more pollution is observed globally and in aquatic environments particularly. However, a recent review of publishing trends in conservation research pointed out the lack of focus on pollution (<2% of publications; Velasco *et al.*, 2015). DEB theory represents an appropriate tool as it was originally developed to evaluate ecotoxicological effects of substances and environmental conditions on organisms, such as the impact of toxicants on physiological traits and the transfer of effects from individuals to populations (Kooijman and Metz, 1984) or the lack of food, extreme temperatures and changes in pH (Kooijman, 1993).

A particular family of DEB models, named DEBtox, was developed to fit the need for simple and effective methods in toxicological tests (Kooijman and Bedaux, 1996). The general idea in the application of DEBtox models is that all compounds within the organism are present in three ranges: too little, enough and too much. Negative effects show up when the compound is either in the too little or too much range (Kooijman, 2018). Stress is linked to the density of harmful compound in the body once it exceeds a no effect concentration (NEC), which is modelled as a change in a DEB model parameter value. Many studies have used DEB modelling to evaluate the effects of stress (e.g. chemical substances) on the life history of various organisms, which may have significant implications for conservation. Klok *et al.* (2014) estimated the impact of petroleum substances on the survival and development of young cod, *Gadus morhua*, which is a critical species exploited in the North Atlantic, where oil and gas drilling activities are extensive. (Kooijman *et al.*, 2009).

### Effect of mixtures

With an exponential increase of organic and chemical substances being released each year (Binetti *et al.*, 2008), concerns about the interactive effects of these substances on the life history of species arise and new studies now aim at evaluating the impact of mixtures on organisms (Baas *et al.*, 2010). Robinson *et al.* (2017) applied a DEBtox mixture model to investigate the effects of semi-chronic binary mixture exposure of insecticides, neocotinoids and fungicides on three bee species (*Apis mellifera*, *Bombus terrestris* and *Osmia bicornis*). The authors identified dominant additive response patterns as well as examples of interactions at small scales (temporal and magnitude-wise), which may need to be accounted for during risk assessments. Grech *et al.* (2019) developed a generic physiologically based toxicokinetic model for rainbow trout (*Onchorhynchus mykiss*), zebrafish (*Danio rerio*), fathead minnow (*Pimephales promelas*) and three-spined stickleback (*Gasterosteus aculeatus*). This model can be used to assess the influence of physiological and environmental factors on the toxicokinetics of chemicals and to provide guidance for assessing their effects in environmental risk assessment.

Conservation efforts generally aim to protect populations or groups of populations, rather than individuals. However, most management strategies in response to toxicant mixtures rely on experimental measurements of the effects of toxicants at the individual level, despite population-level effects not being necessarily proportional to these individual-level responses (Vaugeois *et al.*, 2020). Assuming the position of a manager observing a walleye (*Sander vitreus*) population under stress caused by mixtures of contaminants of emerging concern (CEC) in the Great Lake ecosystem, Vaugeois *et al.* (2021) used a coupled DEB-IBM integrating toxicokinetic-toxicodynamic effects to compare the effectiveness of moderate mitigation of an entire watershed (50% reduction in exposure level) versus intensive mitigation of single river sites (reduction of exposure to a level that does not affect walleye) for three CEC mixture scenarios (agricultural, urban and combined). They observed that small-scale strategies are more effective when focusing on spawning sites and that toxicokinetics are more important to evaluate their effectiveness, while population characteristics are more important to evaluate large-scale strategies.

### Mitigation of harmful effects

Any compound found in excessive quantity (i.e. exceeding the NEC) may become toxic or be considered as pollution. For instance, the increasing demand for seafood products has intensified inputs such as fish feed per unit culture area and, therefore, increased waste generation from aquaculture production systems. Dissolved and particulate organic compounds originating from aquaculture are a potential source of ecosystem degradation and concern for conservation of wild species impacted by it. Therefore, mitigation of these impacts on the environment is needed to ensure the success of conservation efforts. For example, integrated multi-trophic aquaculture systems have gained interest as a mean to optimize nutrient and energy use, to decrease waste, and to diversify fish-farm production. Building on the recent development of detritivorous aquaculture, Galasso *et al.* (2020) used a DEB model to predict the metabolic processes of a ragworm (*Hediste diversicolor*) in various environmental conditions and to estimate its fish waste bioremediation capacity. Another example of such application can be found for the integrated multi-trophic aquaculture of red drum (*Sciaenops ocellatus*) and sea cucumber (*Holothuria scabra*; Chary *et al.*, 2020).

The explicit use of DEB theory to inform the conservation of species particularly sensitive to environmental changes is growing and being recognized, notably by the European Food Safety Authority (More *et al.*, 2019; Spurgeon *et al.*, 2020). Although a DEB approach has yet to be formally applied to crayfish, which are often considered as a sentinel species in freshwater ecosystems, a recent call for a cohesive strategy for the conservation of these animals in the USA stressed the importance of energetic budgets in understanding crayfish growth, population and community dynamics for commercial and non-commercial species (Taylor *et al.*, 2019). Moreover, in a context of increasing anthropic pressures through pollution, habitat degradation (e.g. eutrophication), the use of energetic approaches within Adverse Outcome Pathways frameworks—aimed at the identification of physiological means of action of toxic compounds or diseases on the physiology of organisms—holds much promise for conservation of the diverse array of crayfishes (Taylor *et al.*, 2019).

## Populations and ecosystems

A major challenge with ecosystem-based management approaches to managing natural resources is the vastly different space and time scales that all biological processes operate at: from geological space/time scales to the extremely fast biochemical reactions within individual cells. In fact, the link between different levels of organization is an inherent characteristic of biology. It defines the relationship between the structure and function of ecosystems and has long been a focus in conservation research. While DEB theory formalizes the processes of uptake and use of substrates at the organismal level, the step to the populational level can be done in various ways. Klanjšček *et al.* (2006) used a DEB-structured matrix model to study vital rates and demographic dynamics in populations. Kooi and van der Meer (2010) used a physiological-structured population model to evaluate the evolution of spawning strategies such as the timing of reproduction of the Baltic clam (*Macoma baltica*). Lorena *et al.* (2010) modelled the dynamics of phytoplankton populations using the concept of V1-morphy, a scaling property of DEB theory applicable to organisms replicating by division or for which surface area is proportional to volume (e.g. bacteria, phytoplankton, some macroalgae).

### Individual-based models

Recently, individual-based models (IBM) based on DEB theory may have become the most prolific area for DEB population applications. These IBM are usually agent-based models in which each agent represents an individual of a population. Martin *et al.* (2013) showed how this approach could be used to extrapolate the effect of toxicants measured at the individual level to effects on population dynamics. In a review of animal movement literature, Malishev and Kramer-Schadt (2021) contributed to an important field of conservation physiology by formulating individual energetics in a modelling framework to address the challenges of modelling movement across different scales, species and constraints. Desforges *et al*. (2019) studied the impact of extreme seasonal conditions in the Arctic on the life-history traits of the muskox (*Ovibos moschatus*) and quantified for instance the impact of food limitation on their fecundity. Louati et al. (2020) developed a DEB-IBM to study the size at the time of a sex change and the physiological factors influencing this critical process in dusky groupers (*Epinephelus marginatus*), a species listed as vulnerable by the International Union for Conservation of Nature. Yurek *et al.* (2021) evaluated the restoration potential of self-organizing oyster reefs through a 3D IBM of oyster reef mechanics. Other examples include the coupling of individual DEB models to numerical models of the physico-geochemical characteristics of the environment (Le Goff *et al.*, 2017; Saraiva *et al.*, 2017). This sort of coupling can be particularly useful to infer the impact of future changes on populations as the physical modules describing environmental conditions can be adapted to scenarios of future climatic conditions.

Moreover, as the links between human footprint and ecosystem become more evident, ecosystem-based management tools and methods emerged to include scientific and socioeconomic information into decision making. The aim in this approach is to protect ecosystem structure and functioning, not simply individuals or populations. Integrating individual based DEB models into idealized ecosystem models has yielded insights into how climate change and pollution can act synergistically upon elemental cycling in such systems (Galic *et al.*, 2017). Extending the transfer of scales between organization levels, Forbes *et al.* (2017) linked common ecotoxicological endpoints at the cellular level to chemical impacts on populations and communities and the ecosystem services that they provide. Both studies illustrate how a framework based on mechanistic models that predict impacts on ecosystem services resulting from chemical exposure, combined with economic valuation, can provide a useful approach for informing environmental management.

### Ecosystem models

Ecosystems comprise sets of interacting populations in a given habitat. Most conservation and protection goals relate to quantitative aspects of ecosystem structure and functioning (e.g. biodiversity, resource cycling), making the transfer of scale from the individual to the ecosystem central to conservation research in general. One approach to achieve this transfer of scale is to combine different DEB-based submodels inside a box ecosystem model, as illustrated by Lavaud *et al.* (2020), who used DEB models for an invasive macroalgae and wild and farmed bivalve species to study the impact of aquaculture and eutrophication on the functioning of a coastal ecosystem. A complementary approach is to study the behaviour of simple (un)structured ecosystem models that respect mass and energy conservation principles. For instance, Poggiale *et al.* (2013) evaluated the effects of nutrient input rate on the variability in the dynamics, the functioning and the structure of marine trophic chains (including phytoplankton, zooplankton and a consumer). Maury and Poggiale (2013) formulated a DEB model of the size-structured dynamics of marine communities that integrates mechanistically individual, population and community levels. The authors showed that the simultaneous consideration of individual growth and reproduction, size-structured trophic interactions, the diversity of life-history traits and a density-dependent stabilizing process allow realistic community structure and dynamics to emerge without any arbitrary prescription. This non-exhaustive list of examples shows that DEB applications at higher levels of organization have been carried out in marine ecosystems, often in relation to aquaculture development, carrying capacity and fisheries management. DEB research can therefore constitute a valuable tool to evaluate the impacts of human activities on ecosystems, particularly in the context of species conservation.

## Final remarks

The vast diversity of life on Earth has propelled formalizing the characteristics of all living organisms and placing them within their presumed eco-evolutionary history. The tree of life continues to increase in resolution as we learn more over the centuries. Contrary to physical systems, organisms share some 3.9 billion years of eco-evolutionary history. Scientists can exploit this to better understand biological processes in time. DEB theory offers a lens by which to view organisms in a comparable way, which helps to understand the underlying metabolic properties by which they differ (Marques *et al.*, 2018). The physiological mechanisms of energy intake and allocation have not been so commonly explored and modelled for conservation purposes, certainly not in a context where a comparable model for metabolism is applied such that species differ only in terms of parameter values. The complexity of metabolic processes, which determine life history trade-offs and a historical focus on the effect of exogenous factors in the study of life history evolution may explain these circumstances (Teixeira, 2016). However, with a unifying formalism accounting for various scales of time and organization, we illustrated how DEB theory can remediate this situation and provide a new scope to the field of conservation.

In addition to the applications described in this paper, exciting new developments of the theory include sub-organismal disciplines such as genomics and proteomics, with research on inter-individual genetic (Sadoul *et al.*, 2020) and phenotypic variability (Mariño et al., 2018), an area yet to be fully assimilated in DEB models. Conservation studies have already embraced these new techniques and benefitted from their application. Nonetheless, DEB theory provides the theoretical background for bridging this gap and new applications at the cellular level are emerging (Murphy *et al.*, 2018). The upcoming *Seventh International Symposium on DEB theory: Forecasting in a Changing World* will showcase many contributions that could provide valuable insight into conservation issues. This special issue aims to compile cutting edge contributions at the crossroad between DEB Theory and Conservation Physiology.
